# Supplementary material for: A novel L1CAM isoform with angiogenic activity generated by NOVA2-mediated alternative splicing
Source: eLife. 2019 Mar 4;8:e44305. doi: 10.7554/eLife.44305 (PMC6398979; doi:10.7554/eLife.44305)
Supplement: Supplementary file 1. [file elife-44305-supp1.docx]

**Supplementary Table 1. Primers used in RT-PCR, RT-qPCR and cloning experiments.**

| **Primers used in RT-PCR experiments** | **Sequence (5’-3’)** |
| --- | --- |
| hL1E23_F | CACAGCGGGTGAAAACTACA |
| hL1E28_R | GCATCTCCTGTCCTGGACTC |
| mL1E23_F | AGCCACAGCAGGTGAAAACT |
| mL1E28_R | CATGGCTGGACCTTGCTATT |
| hL1E24_F | GCCTGACACTGACTACGAGA |
| hL1E26_R | CCTGGGTGTCCTCCTTATCC |
| p-L1_F | GGAATTCAAGGGAAAGTGAGCCCTGAT |
| BGH_R | TAGAAGGCACAGTCGAGG |
| mL1E1_F | CAAGATGGTCGTGATGCTGC |
| mL1E3_R | CGTGATGACAGGTGGCTCTA |
| mL1E26_F | TGAAGGACAAGGAGGACACT |
| mL1E28'_R | CCAAAGGCCTTCTCTTCATTG |
| mhGAPDH_F | TCAAGAAGGTGGTGAAGCAGG |
| mhGAPDH_R | ACCAGGAAATGAGCTTGACAAA |
| hL1E1_F | TGCTTATCCAGATCCCCGAG |
| hL1E3_R | TCTGTGGGGAAGACAACCAG |
| hL1E26_F | GGCCCGACCGATGAAAG |
| hL1E28'_R | TTGATGTCCCCGTTGAGC |
| **Primers used in RT-qPCR experiments** | **Sequence (5’-3’)** |
| mNova2_F | AGGACTGATCATCGGTAAGG |
| mNova2_R | GGGTCTTCCTGTACCTTCTG |
| hNova2_F | CAGCTTTATTGCCGAGAAGG |
| hNova2_R | ACCCATGCTCCTGACTGTTC |
| mhGAPDH_F | TCAAGAAGGTGGTGAAGCAGG |
| mhGAPDH_R | ACCAGGAAATGAGCTTGACAAA |
| mUbb_F | TCTTCGTGAAGACCCTGACC |
| mUbb_R | CAGGTGCAGGGTTGACTCTT |
| CLIP-E2_F | ATTGGGACTGCTCTCAGCAT |
| CLIP-E2_R | CCCAATCCTCCAACTTACCA |
| CLIP-I2_F | TCTGCTTCTGTTGGAATTTCTG |
| CLIP-I2_R | ACCCCCAAAAGAAGAGCAAT |
| CLIP-E25_F | GCCCTGTGCGAGTTTCTACT |
| CLIP-E25_R | AGTATTTGCCACCCTTGCTG |
| CLIP-E26_F | CTCCTCTGGTATGTCTGTAGTGAA |
| CLIP-E26_R | TGTCCCTGCTCACCTGTACTC |
| CLIP-I26_R | CAGTTCTGCTTGACCTTTGCT |
| CLIP-I26_R | GACATCCCTACTGTCACATCTTACC |
| CLIP-I27_F | GGGCCCTGCAATACATCTAC |
| CLIP-I27_R | GATGTCTCCGTTGAGAGATGG |
| **Primers used in cloning experiments** | **Sequence (5’-3’)** |
| mNova2_F | AGGACTGATCATCGGTAAGG |
| mNova2_R | GGGTCTTCCTGTACCTTCTG |
| hNova2_F | CAGCTTTATTGCCGAGAAGG |
| hNova2_R | ACCCATGCTCCTGACTGTTC |
| mhGAPDH_F | TCAAGAAGGTGGTGAAGCAGG |
| mhGAPDH_R | ACCAGGAAATGAGCTTGACAAA |
| mUbb_F | TCTTCGTGAAGACCCTGACC |
| mUbb_R | CAGGTGCAGGGTTGACTCTT |
| CLIP-E2_F | ATTGGGACTGCTCTCAGCAT |
| CLIP-E2_R | CCCAATCCTCCAACTTACCA |
| CLIP-I2_F | TCTGCTTCTGTTGGAATTTCTG |
| CLIP-I2_R | ACCCCCAAAAGAAGAGCAAT |
| CLIP-E25_F | GCCCTGTGCGAGTTTCTACT |
| CLIP-E25_R | AGTATTTGCCACCCTTGCTG |
| CLIP-E26_F | CTCCTCTGGTATGTCTGTAGTGAA |
| CLIP-E26_R | TGTCCCTGCTCACCTGTACTC |
| CLIP-I26_R | CAGTTCTGCTTGACCTTTGCT |
| CLIP-I26_R | GACATCCCTACTGTCACATCTTACC |
| CLIP-I27_F | GGGCCCTGCAATACATCTAC |
| CLIP-I27_R | GATGTCTCCGTTGAGAGATGG |
